# From half-metallic to magnetic semiconducting triazine g-C_4_N_3_: computational designs and insight

**DOI:** 10.1039/d1ra05348e

**Published:** 2021-12-07

**Authors:** Pham Nam Phong, Nguyen Thi Ngoc, Pham Thanh Lam, Manh-Thuong Nguyen, Huy-Viet Nguyen

**Affiliations:** School of Engineering Physics, Hanoi University of Science and Technology (HUST) 1 Dai Co Viet Road Hanoi Vietnam phong.phamnam@hust.edu.vn +84 24 3869 3498 +84 24 3869 3350; Institute of Physics, Vietnam Academy of Science and Technology (VAST) 10 Dao Tan Street Hanoi Vietnam

## Abstract

We have given, for the first time, physicochemical insight into the electronic structure routes from half-metallic to magnetic semiconducting triazine g-C_4_N_3_. To this end, three material designs have been proposed using density functional calculations. In one design, this half-metal is first made semiconducting *via* hydrogenation, then tailored with B and N atomic species, which gives a new prototype of the antiferromagnetic semiconductor monolayer HC_4_N_3_BN. In the others, it can be rendered spin gapless semiconducting with H and B or C, followed by F or O tailoring, which eventually leads to the two new bipolar ferromagnetic semiconductors HC_4_N_3_BF and HC_4_N_3_CO. These monolayers are considered to be novel materials in spintronics.

## Abstract 2

A simple picture of charge transfer has been given, for the first time, as physicochemical insight into the electronic structure routes from half-metallic to magnetic semiconducting triazine g-C_4_N_3_.

## Graphical abstract

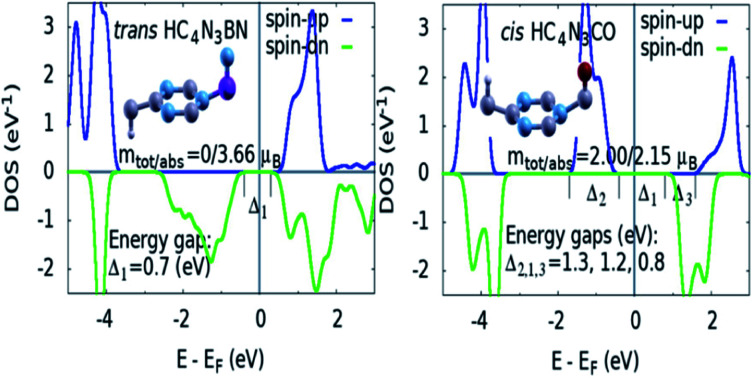

## 1 Introduction

Half-metals and magnetic semiconductors are interesting materials and promising candidates for spintronics.^1–3^ The band structure of half-metals (HMs) shows the coexistence of metallic nature in one spin state and semiconducting nature in the other.^4^ This signature avails them in essential applications such as spin-polarized current generation and injection.^5^ Simply put, magnetic semiconductors (MSs) are semiconductors endowed with magnetism.^1,3^ Early investigations aimed to make non-magnetic semiconductors ferromagnetic and this has been actively explored from dilute magnetic semiconductors^6,7^ to nanocomposite semiconductor-ferromagnets.^8^ In view of recent studies on emerging two-dimensional (2D) and layered MSs,^9–11^ particularly from computational perspectives, it is desirable to design new monolayers of these materials.

Spin gapless semiconductors (SGSs), since their inception,^12,13^ have received growing interest toward next-generation spintronic devices.^14–16^ Their unique band structure, featuring fully spin-polarized carriers of zero excitation energy, bridges the half-metallic and magnetic semiconducting or gapless material classes.^13,16^ Another piece fitting into the picture is gapless half-metals (GHMs).^17^ The semiconducting spin state of a HM becomes zero-gap in a GHM, similar to the gap opening with its metallic counterpart in SGSs.^18^ The band structure of such gapless systems is extremely sensitive to external influences, and gapless states could exist in between semiconducting and metallic ones in certain cases.^12,13^ It is the distinctive band structures of those material classes that allow tuning of the system from one state to another under external parameters, such as strain, structure or composition, and surface engineering.^12,15^ This guides us in the search for electronic structure routes in designing MS monolayers from a HM prototype.

Our starting HM is triazine g-C_4_N_3_,^19,20^ a member of the graphitic carbon nitride (g-CN) material family. Strain engineering has been shown to effectively induce and regulate magnetism or cause structural transitions in these 2D lattices.^21–23^ In addition, different structures were tailored for them with such novel properties as previously reported.^22–26^ Here, using density functional theory (DFT) calculations, we propose different material designs to tailor this g-CN by H and 2p elements. Eventually, this has given us three new monolayers of MS, one being antiferromagnetic, which is central to the emerging subfield of antiferromagnetic spintronics,^27,28^ while the other two are bipolar magnetic semiconducting,^29^ with useful spin currents with reversible polarization. Among our sought-after electronic structure routes from HM to MS, we have found notable routes *via* SGS/GHM states. Finally, physicochemical insights into this finding will be given through a simple picture of charge transfer.

## 2 Method and computational details

**Fig. 1:**
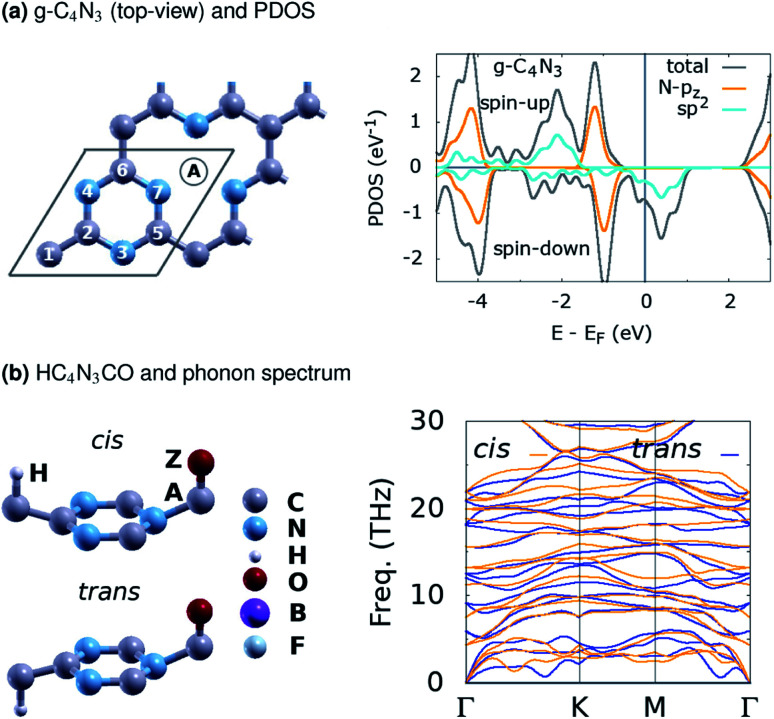
(a) The structure of g-C_4_N_3_, with the rhombus marking its (1 × 1) unit cell, plus the A-atom at the vacant site, and the PDOS. (b) One proposed HC_4_N_3_AZ material design with a phonon spectrum.

In this study, various g-CN-based material designs are proposed computationally as HC_4_N_3_AZ, see Fig. 1. Here, A is the first atom (B, C, Li) tailored at the g-CN lattice vacant site, Z the second atom (N, F, O) upon the first one, and H the hydrogen atom upon C(1). In addition, HC_4_N_3_AZ is considered in its *cis*- or *trans*-configuration, as shown, where H and Z atoms are on the same side or opposite sides of the C_4_N_3_A lattice, respectively. For every HC_4_N_3_AZ structure under consideration, there is a related ZC_4_N_3_AH structure in which the sites of the H and Z atoms are exchanged. The local geometry around these sites somewhat resembles other monolayers.^30–32^

We have carried out plane-wave DFT calculations implemented in the Quantum ESPRESSO suite,^33^ using PBEsol and HSE functionals.^34,35^ A kinetic energy cutoff is chosen at 80 Ry with the optimized norm-conserving Vanderbilt pseudopotentials.^36^ The unit cells are relaxed against the force and pressure convergence thresholds of 10^−3^ a.u. and 3 kbar, respectively. All subsequent calculations have been performed on top of the PBEsol relaxed structures, with that functional for the phonon spectra, while the HSE hybrid one is used for other properties. The vacuum layer is about 19 Å in thickness, justified to simulate our 2D systems. Brillouin zone sampling is checked against the optimal (12 × 12) *k*-point grid with suitable smearing for (1 × 1) periodicity, to converge the total energies within 1 mRy. Denser *k*-grids of (24 × 24) are used in non-self-consistent and phonon calculations. Structures and spin/bonding charge density plots are rendered using the XCrySDen^37^ and VESTA^38^ programs. We use an implementation of the Bader analysis for partial atomic charge estimation.^39^

## 3 Results and discussion

Let us first present the material designs and properties in Section 3.1. We start off by clarifying the half-metallic ferromagnetic nature of pristine g-C_4_N_3_, then consider the magnetism of our designed materials. That gives the key to those designs, which is obviously the chemistry of the H-atom at C(1) and various tailored A-atoms at the vacant sites of this gCN lattice. It has radically changed the electronic and magnetic properties of the system and justified such elaborate designs in our proposal. In Section 3.2, we discuss the related electronic structures and physicochemical insight, through a simple charge transfer analysis.

### 3.1 Material designs and properties

Table 1 summarizes the basic structural and energetic properties of g-C_4_N_3_ and three proposed material designs, namely HC_4_N_3_BN, HC_4_N_3_BF and HC_4_N_3_CO. First, we note that the explored properties are highly related with their structures. Specifically, the configurations ZC_4_N_3_AH, with the H and Z atomic sites exchanged, are non-magnetic and not considered afterwards. This configuration is less favorable in energy than its HC_4_N_3_AZ counterpart, except for the case of *trans*-HC_4_N_3_BN, see table column Δ*E*_HZ_. These elaborate designs are inspired by one of the very first atomically controlled hydrogenation studies on tailoring graphene magnetism,^40^ as well as a recent first-principles investigation on 2D carbon nitride nanosheets, already synthesized or theoretically predicted.^41^ Moreover, our study concerns electronic properties and magnetism as in ref. 41, so any kinetics related problem is beyond its scope, and we propose these designs without considering atomic or molecular dissociation/diffusion processes thereon. Instead, a brief check on their dynamical stability is done with the numbers of imaginary phonon frequencies *n*_if_ at the *Γ*/*K*/*M* points, given in the table. A phonon spectrum without imaginary frequencies in Fig. 1(b) for HC_4_N_3_CO signifies the dynamical stability of this 2D lattice.

**Table 1:** Structural and energetic properties, with classifications of g-C_4_N_3_ and three proposed material designs, *cf.*Fig. 1(a), 2(b, d, f) and Table 2. The results are the lattice constant *a* (Å), numbers of imaginary phonon frequencies *n*_if_ at the *Γ*/*K*/*M* points, energy separation Δ*E*_HZ_ (eV) between the HC_4_N_3_AZ and ZC_4_N_3_AH structures and magnetic energy Δ*E*_m_ (eV)

System	*a*	*n*_if_	Δ*E*_HZ_ a	Δ*E*_m_ b	Class^c^
g-C_4_N_3_	4.83*	0/0/0		−0.41	HM-FM

HC_4_N_3_BN					
*cis*	4.88	0/0/0	−0.18	−1.15	MS-AFM
*trans*	4.89		0.29	−1.24	

HC_4_N_3_BF					
*cis*	4.88	0/0/0	−1.88	−0.93	BMS-FM
*trans*	4.89		−1.63	−0.84	

HC_4_N_3_CO					
*cis*	4.83	0/0/0	−1.52	−0.91	BMS-FM
*trans*	4.85		−1.72	−0.86	

a Δ*E*_HZ_ = *E*(HC_4_N_3_AZ) − *E*(ZC_4_N_3_AH).

b Δ*E*_m_ = *E*(magnetic) − *E*(non-magnetic).

c HM/MS/BMS: half-metal/(bipolar) magnetic semiconductor; FM/AFM: ferromagnet/antiferromagnet; *4.84.^20^

**Fig. 2:**
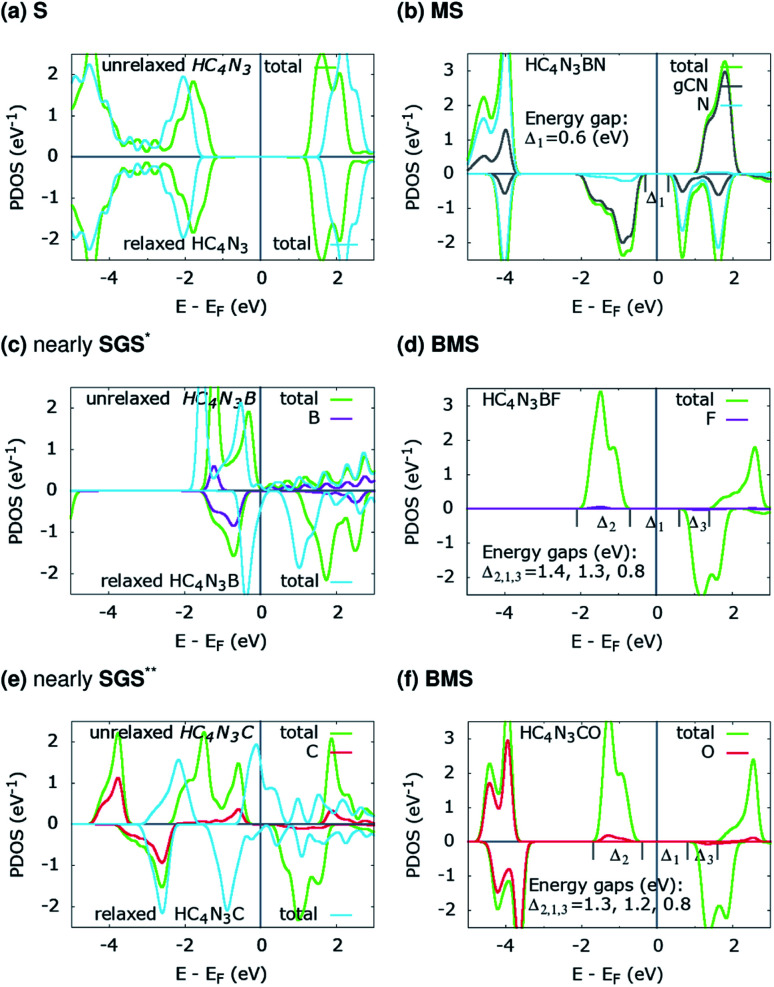
PDOS of the three proposed material designs. The unrelaxed configurations (a, c and e) are from their relaxed counterparts (b, d and f) by removing the respective atoms, see text and Table 2. *small DOS near *E*_F_ for spin-up states; **small-gap BMS.

The projected density of states (PDOS) is presented for pristine g-C_4_N_3_ in Fig. 1(a) and the three designs in Fig. 2(b, d and f). The differences in the PDOS of their *cis*- and *trans*-configurations are not radical, despite a structural contrast. In addition, there is a considerable increase in the magnetic energy Δ*E*_m_, see that column in Table 1, given as the energetic separation between the magnetic and non-magnetic states. A closer look at Fig. 1(a) reveals that, consistent with ref. 20, the HM and FM properties of g-C_4_N_3_ are mostly from pyridinic N-sp^2^ electrons, one of them is left unpaired in the unit cell making its magnetization 1 *μ*_B_. Interestingly, C_4_N_3_A is a non-magnetic semiconductor for H or Li as the A-atoms, the latter reported previously,^42^ whereas when the A-atom is C or B the material becomes metallic. Both isoelectronic HC_4_N_3_BF and HC_4_N_3_CO are BMS-FM in nature, while HC_4_N_3_BN is a MS-AFM, see Table 1 footnotes, Fig. 2(b, d, f) and Table 2.

**Table 2:** Bader analyses for the respective designs in Fig. 2. Here, *q̄*_3C_, *q̄*_3N_, *q*_A_ and *q*_Z_ are the average charges of the C(2,5,6) and N(3,4,7) atoms and the charges of the A-, and Z- atomic species. The total and absolute magnetization are in *μ*_B_ per unit cell

System^a^	*q̄*_3C_	*q̄*_3N_	*q*_A_	*q*_Z_	*m*_tot_	*m*_abs_	Class^b^
*HC_4_N_3_*	2.80	−2.83			0.00	0.00	S
HC_4_N_3_BN	2.48	−3.14	3.00	−1.19	0.00	3.65	MS
*HC_4_N_3_B*	2.22	−3.09	2.53		1.00	2.08	Nearly SGS
HC_4_N_3_BF	2.29	−3.02	3.00	−0.98	2.00	2.15	BMS
*HC_4_N_3_C*	2.18	−2.46	0.60		2.00	2.12	Nearly SGS
HC_4_N_3_CO	2.22	−2.50	2.57	−2.05	2.00	2.15	BMS
HC_4_N_3_B	1.96	−3.01	3.00		1.08	1.35	Nearly GHM*
HC_4_N_3_C	1.64	−2.37	2.05		−0.49	0.61	

a Italics denoting the unrelaxed systems.

b S/MS/BMS/SGS/GHM: semiconductor/(bipolar) magnetic/spin gapless semiconductor/gapless half-metal *small spin-up/down DOS near *E*_F_, see Fig. 2(c and e).

**Fig. 3:**
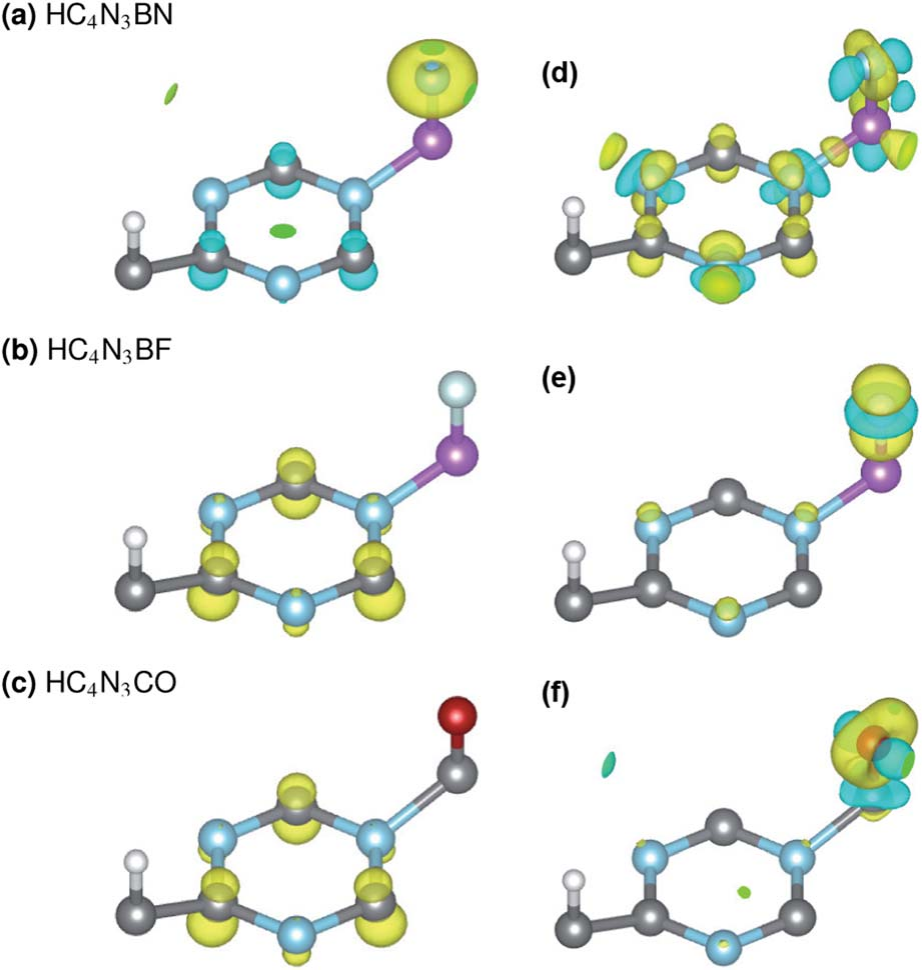
Spin *ρ*_s_ (left) and bonding charge* *ρ*_b_ (right) densities of the three proposed material designs, with positive/negative values in yellow/cyan and isosurface levels of 0.04 and 0.02, respectively. *ρ_b_ = *ρ*(*M*–*X*) − *ρ*(*M*) − *ρ*(*X*), where *M* is BN, F and O, while *X* is HC_4_N_3_, HC_4_N_3_B and HC_4_N_3_C.

Each unrelaxed system with a PDOS in Fig. 2(a, c and e) is obtained by removing the respective tailored atoms from its relaxed counterpart (b, d and f). The unrelaxed (c) and (e) are strained, nearly SGS in nature, as in their respective PDOS graphs, and under structural relaxation they become metallic, or precisely a nearly GHM, see the PDOS graphs and Table 2 footnotes, which clearly signifies the bandgap engineering by strain in 2D materials.^21–23,43^ They serve as some intermediate steps of these designs and help in clarifying the electronic structure routes, as follows. In Table 2, *q̄*_3C_, *q̄*_3N_, *q*_A_ and *q*_Z_ denote the average charges of the C(2,5,6) and N(3,4,7) and the charges of the A- and Z-atoms. Fig. 3 shows the spin *ρ*_s_ and bonding charge *ρ*_b_ densities. The latter is given as *ρ*_b_ = *ρ*(*M*–*X*) − *ρ*(*M*) − *ρ*(*X*), the electron density difference between the *M*–*X* system and its sublattices.

### 3.2 Electronic structures and physicochemical insight

On the electronic structure route of S-to-MS, for the design in Fig. 2(a, b) and 3(a), all three valence electrons of B are transferred to the N and HC_4_N_3_ sublattices, *cf.*Table 2, second row. The former receives 1.2*e*, whereas 1.8*e* is divided almost evenly among those six atoms in the hexagonal ring of the latter, leaving its C(1) and H atoms virtually unaffected, see Fig. 3(a) right plot. Notably, the charge is transferred to such two opposite spin polarized sublattices, see the PDOS peaks around −4 and −1 eV and Fig. 3(a) left plot, making their spin magnetic moments about 1.8 units each. We note the total/absolute magnetization values of 0.00/3.65 in the table. This tailoring by B plus N therefore endows our semiconductor with antiferromagnetism. It is worth referring to the study on 3d^(*m*,*n*)^ transition metal pairs doped half-metallic dilute (anti)ferromagnetic semiconductors (*m* + *n* = 10).^44^ Here, the new material HC_4_N_3_BN gives another prototype of MS-AFM, in particular a monolayer one *per se*, with 2p-non-metals replacing metal dopants.

The S-to-MS route is also viable for the design in Fig. 2(d). The difference lies in the fact that nearly 1*e* goes to F, Table 2 fourth row, making its spin magnetic moment practically zero, see the F-channel in Fig. 2(d). Meanwhile, the remaining charge is sent off to the HC_4_N_3_ sublattice, see the PDOS peak below the Fermi energy there and Fig. 3(b) left plot as well, rendering a magnetization of 2 units. Unlike the preceding AFM, in this case our material is a monolayer FM.

The electronic structure route of SGS-to-BMS is clearer for the design in Fig. 2(e and f) than for the one in Fig. 2(c), which is also less clear than the S-to-MS route discussed above. Upon tailoring by O in the former, there are only minor amounts of charge rearranged within the HC_4_N_3_ sublattice, hence its magnetization has hardly changed, *cf.*Table 2 fifth and sixth rows. We merely observe such a major charge of 2*e* transferred from C to O, see also the C-channel PDOS peak below the Fermi energy in Fig. 2(e) and the densities in Fig. 3(c). This undoubtedly complies with the opening of a *Δ*_1_ energy gap, which was reported for other systems in ref. 29 and 43, leading to a BMS.

In the latter design, the 0.98*e* moved to F is in fact from B, 0.47*e*, C(2,5,6) plus N(3,4,7), 0.42*e*, and H, 0.09*e*, given in Table 2 third and fourth rows. This charge transfer adheres to the energy gap opening, as seen in Fig. 2(c, d) and overall adds 1 unit to the magnetization. Given these charge analyses, the bonding between F or N and B, *i.e.* the HC_4_N_3_B sublattice, is somewhat ionic in nature, whereas in the HC_4_N_3_CO unit cell there is a C–O dative bond, as is present in carbon monoxide.

## 4 Conclusions

We close this work with some outlooks. The electronic structure of triazine g-C_4_N_3_ is explored using DFT calculations, where three material designs have been proposed to make this half-metallic gCN magnetic semiconducting. In one route from half-metal to magnetic semiconductor, hydrogenation and B plus N tailoring lead to one material design HC_4_N_3_BN, which is a new prototype of an antiferromagnetic semiconductor monolayer. In the others, two designs with tailored atomic species B and F, or C and O, give the new bipolar ferromagnetic semiconductors HC_4_N_3_BF and HC_4_N_3_CO, respectively.

In view of the studies on gCN thin-films^45^ or van der Waals antiferromagnets,^9^ as well as novel heterojunctions of 2D transition metal chalcogenides,^46^ computationally designing layered materials or structures from these monolayer magnetic semiconductors is promising. Further investigations on their stability, dynamics, transport and magnetism are therefore highly demanded. It is also advisable to consider the spin–orbit coupling, which has been proven to be significant for certain g-CN systems, in search of metal-free 2D topological insulator states.^47^ The main idea behind our study, with its results illustrated by means of charge transfer, will be helpful to the prospective, especially experimental, works on related nanomaterials, as well as towards more practical applications in spintronics.

## Conflicts of interest

There are no conflicts to declare.

## Supplementary Material

## References

[cit1] Felser C., Fecher G., Balke B. (2007). Angew. Chem., Int. Ed..

[cit2] Žutić I., Fabian J., Das Sarma S. (2004). Rev. Mod. Phys..

[cit3] Wolf S. A., Awschalom D. D., Buhrman R. A., Daughton J. M., von Molnár S., Roukes M. L., Chtchelkanova A. Y., Treger D. M. (2001). Science.

[cit4] Katsnelson M. I., Irkhin V. Y., Chioncel L., Lichtenstein A. I., de Groot R. A. (2008). Rev. Mod. Phys..

[cit5] Hirohata A., Takanashi K. (2014). J. Phys. D: Appl. Phys..

[cit6] Dietl T., Ohno H. (2014). Rev. Mod. Phys..

[cit7] Sato K. (2010). et al.. Rev. Mod. Phys..

[cit8] Dietl T. (2010). Nat. Mater..

[cit9] Miao N., Xu B., Zhu L., Zhou J., Sun Z. (2018). J. Am. Chem. Soc..

[cit10] Li X., Yang J. (2019). J. Am. Chem. Soc..

[cit11] Qin T., Wang Z., Wang Y., Besenbacher F., Otyepka M., Dong M. (2021). Nano-Micro Lett..

[cit12] Wang X. L. (2008). Phys. Rev. Lett..

[cit13] Ouardi S., Fecher G. H., Felser C., Kübler J. (2013). Phys. Rev. Lett..

[cit14] Wang X., Cheng Z., Zhang G., Yuan H., Chen H., Wang X.-L. (2020). Phys. Rep..

[cit15] Rani D., Bainsla L., Alam A., Suresh K. G. (2020). J. Appl. Phys..

[cit16] Yue Z., Li Z., Sang L., Wang X. (2020). Small.

[cit17] Du Y., Xu G. Z., Zhang X. M., Liu Z. Y., Yu S. Y., Liu E. K., Wang W. H., Wu G. H. (2013). Europhys. Lett..

[cit18] Gao G. Y., Yao K.-L. (2013). Appl. Phys. Lett..

[cit19] Lee J. S., Wang X., Luo H., Dai S. (2010). Adv. Mater..

[cit20] Du A., Sanvito S., Smith S. C. (2012). Phys. Rev. Lett..

[cit21] Liu L. Z., Wu X. L., Liu X. X., Chu P. K. (2015). Appl. Phys. Lett..

[cit22] Li X., Zhang S., Wang Q. (2013). Phys. Chem. Chem. Phys..

[cit23] Li X., Zhou J., Wang Q., Kawazoe Y., Jena P. (2013). J. Phys. Chem. Lett..

[cit24] Zhang X., Zhao M., Wang A., Wang X., Du A. (2013). J. Mater. Chem. C.

[cit25] Zhang X., Wang A., Zhao M. (2015). Carbon.

[cit26] Pan H., Zhang H., Sun Y., Li J., Du Y., Tang N. (2017). Phys. Rev. B.

[cit27] Baltz V., Manchon A., Tsoi M., Moriyama T., Ono T., Tserkovnyak Y. (2018). Rev. Mod. Phys..

[cit28] Jungwirth T., Marti X., Wadley P., Wunderlich J. (2016). Nat. Nanotechnol..

[cit29] Li X., Wu X., Li Z., Yang J., Hou J. G. (2012). Nanoscale.

[cit30] Xu B., Yin J., Xia Y. D., Wan X. G., Liu Z. G. (2010). Appl. Phys. Lett..

[cit31] Wu P., Huang M., Cheng W., Tang F. (2016). Phys. E.

[cit32] Luo M., Xu Y. E., Song Y. X. (2018). J. Supercond. Novel Magn..

[cit33] Giannozzi P. (2017). et al.. J. Phys.: Condens. Matter.

[cit34] Perdew J. P., Ruzsinszky A., Csonka G. I., Vydrov O. A., Scuseria G. E., Constantin L. A., Zhou X., Burke K. (2008). Phys. Rev. Lett..

[cit35] Heyd J., Scuseria G. E., Ernzerhof M. (2006). J. Chem. Phys..

[cit36] Hamann D. R. (2013). Phys. Rev. B: Condens. Matter Mater. Phys..

[cit37] Kokalj A. (2003). Comput. Mater. Sci..

[cit38] Momma K., Izumi F. (2011). J. Appl. Crystallogr..

[cit39] (b) http://theory.cm.utexas.edu/bader/

[cit40] González-Herrero H., Gómez-Rodríguez J. M., Mallet P., Moaied M., Palacios J. J., Salgado C., Ugeda M. M., Veuillen J.-Y., Yndurain F., Brihuega I. (2016). Science.

[cit41] Bafekry A., Shayesteh S. F., Peeters F. M. (2019). J. Appl. Phys..

[cit42] Hashmi A., Hu T., Hong J. (2014). J. Appl. Phys..

[cit43] Ding Y., Wang Y. (2013). Appl. Phys. Lett..

[cit44] Akai H., Ogura M. (2006). Phys. Rev. Lett..

[cit45] Hashmi A., Farooq M. U., Hu T., Hong J. (2015). J. Phys. Chem. C.

[cit46] Li F., Tao R., Cao B., Yang L., Wang Z. (2021). Adv. Funct. Mater..

[cit47] Wang A., Zhang X., Zhao M. (2014). Nanoscale.

